# Genome-Wide Assessment of the Association of Rare and Common Copy Number Variations to Testicular Germ Cell Cancer

**DOI:** 10.3389/fendo.2013.00002

**Published:** 2013-01-29

**Authors:** Daniel Edsgärd, Marlene D. Dalgaard, Nils Weinhold, Agata Wesolowska-Andersen, Ewa Rajpert-De Meyts, Anne Marie Ottesen, Anders Juul, Niels E. Skakkebæk, Thomas Skøt Jensen, Ramneek Gupta, Henrik Leffers, Søren Brunak

**Affiliations:** ^1^Department of Systems Biology, Technical University of DenmarkLyngby, Denmark; ^2^Department of Growth and Reproduction, RigshospitaletCopenhagen, Denmark

**Keywords:** copy number variation, rare variants, testicular germ cell cancers

## Abstract

Testicular germ cell cancer (TGCC) is one of the most heritable forms of cancer. Previous genome-wide association studies have focused on single nucleotide polymorphisms, largely ignoring the influence of copy number variants (CNVs). Here we present a genome-wide study of CNV on a cohort of 212 cases and 437 controls from Denmark, which was genotyped at ∼1.8 million markers, half of which were non-polymorphic copy number markers. No association of common variants were found, whereas analysis of rare variants (present in less than 1% of the samples) initially indicated a single gene with significantly higher accumulation of rare CNVs in cases as compared to controls, at the gene *PTPN1* (*P* = 3.8 × 10^−2^, 0.9% of cases and 0% of controls). However, the CNV could not be verified by qPCR in the affected samples. Further, the CNV calling of the array-data was validated by sequencing of the *GSTM1* gene, which showed that the CNV frequency was in complete agreement between the two platforms. This study therefore disconfirms the hypothesis that there exists a single CNV locus with a major effect size that predisposes to TGCC. Genome-wide pathway association analysis indicated a weak association of rare CNVs related to cell migration (false-discovery rate = 0.021, 1.8% of cases and 1.1% of controls). Dysregulation during migration of primordial germ cells has previously been suspected to be a part of TGCC development and this set of multiple rare variants may thereby have a minor contribution to an increased susceptibility of TGCCs.

## Introduction

Testicular germ cell cancer (TGCC) is the most common malignancy in young men aged 15−45 years. The incidence has increased over the last decades and is highest in the Nordic countries with 8–9 cases per 100,000, whereas the incidence in men of African and Asian ancestry is five-fold lower (Chia et al., 2010). Environmental exposure partly explains the increasing incidence and ancestral disparity (Skakkebaek et al., 2001), but there is also evidence of a substantial genetic contribution to TGCC susceptibility. The familial aggregation of TGCC is one of the highest among cancers. Brothers and sons of TGCC patients have an 8–10 times and 4–6 times higher risk to develop the disease, while the risk increases 75- and 35-fold for monozygotic and dizygotic twins, respectively (Swerdlow et al., 1997; Hemminki and Li, 2004). Despite the relatively high degree of heritability, genome-wide familial linkage analyses have not identified any loci predisposing for TGCC, and candidate studies have only found one rare deletion (2–3%) at the Y chromosome that confers a modest 2–3 fold increased risk (Nathanson et al., 2005). Recently, genome-wide association studies that search for common single nucleotide variants associated to TGCC have identified susceptibility loci at the genes *KITLG*, *SPRY4*, *BAK1*, *DMRT1*, *TERT*, and *ATF7IP* (Kanetsky et al., 2009; Rapley et al., 2009; Turnbull et al., 2010; Dalgaard et al., 2012). The strongest association was found at *KITLG* with a greater than 2.5-fold increased risk of disease. Consistent with the relatively high familial relative risk, this is the largest effect size found for any single loci among cancers. However, a considerable portion of the heritability remains to be explained.

Here we investigate constitutional DNA copy number variations (CNVs) as another source of genetic variability that may contribute to the development of TGCC. Recent studies have described associations of common CNVs with neuroblastomas (Diskin et al., 2009), systemic autoimmunity (Fanciulli et al., 2007), psoriasis (Hollox et al., 2007), and osteoporosis (Yang et al., 2008). Rare variants, typically originating from recent and *de novo* events, constitute a significant portion of genomic variation. The thousand genomes project indicates that there are about 20,000 CNVs with allele frequencies down to 1% (1000 Genomes Project Consortium, 2010; Mills et al., 2011). The contribution of such rare, or even rarer, variants, to complex disease susceptibility is to a large extent unknown, but they seem to play an important role in psychiatric disorders (International Schizophrenia Consortium, 2008; Pinto et al., 2010) and they have been indicated to influence childhood obesity (Glessner et al., 2010). Further, identification of *de novo* mutations is possible in studies of family-trios and recently three *de novo* CNVs were found in 3 out of 43 TGCC trios, a frequency higher than found in two other cancer types (Stadler et al., 2012), highlighting the paradigm of rare genetic events influencing susceptibility to TGCC. To date, case-control association studies of individual rare CNVs have insufficient power to identify disease-causing variants. To evaluate the impact of rare CNVs with respect to risk for TGCC, we therefore compared the genome-wide burden of rare CNVs and investigated whether any genes or pathways were targeted by multiple rare CNVs such that their aggregated frequency was higher in cases than in controls.

In summary, to assess the effect of CNVs on TGCC we genotyped a Danish case-control cohort (Dalgaard et al., 2012) and analyzed the resulting data with respect to the association of both common and rare germline CNVs to TGCC.

## Results

**Figure 1:**
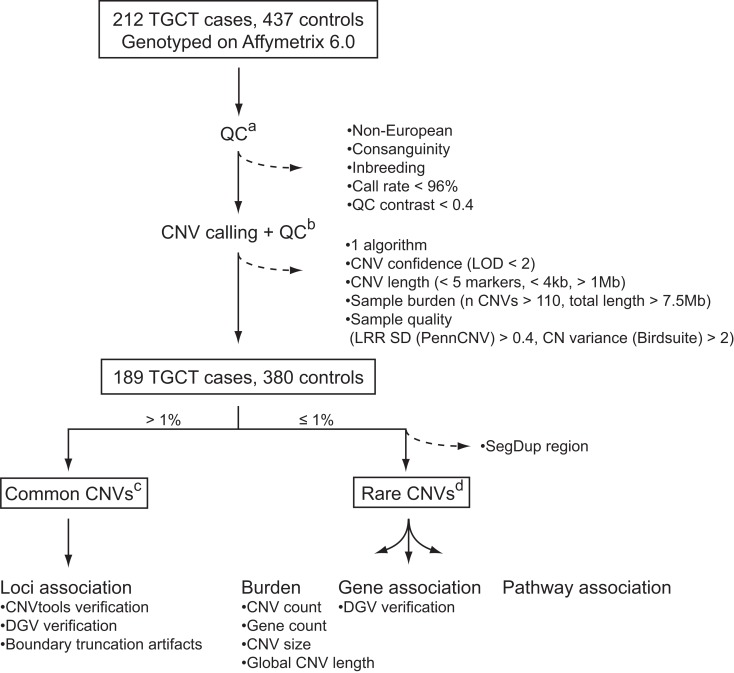
**CNV quality control and analysis**. Dashed arrows indicate CNVs and samples that were excluded from the analysis: (a) quality control of samples based on SNP calls; (b) quality control of CNVs and samples based on CNV calls; (c) association analysis of common CNVs; (d) association analysis of rare CNVs with respect to genomic burden, as well as genes and pathways with an excess of rare CNVs among cases. See “Materials and Methods” for further details. LOD, log odds; LRR SD, log R ratio standard deviation; DGV, database of genomic variants.

To identify CNVs that confer a risk to TGCC, we analyzed common and rare variants in a genome-wide dataset of approximately 1.8 million markers in a Danish cohort constituting 212 TGCC cases and 437 controls. Application of stringent quality control criteria for reliable CNV identification (Figure 1) resulted in a final discovery set of 189 cases and 380 controls. Common variants were defined as CNVs present in more than 1% of the study population, and rare variants as CNVs present in no more than 1% of the studied subjects. Common variants were analyzed with respect to individual locus association, and rare variants with respect to overall genetic burden, gene association, and pathway association.

### Locus association analysis

In order to identify common CNVs associated with TGCC, binary copy number state frequencies of the case and control cohorts were compared at all loci with CNV frequencies above 1%. We observed one genome-wide significant deletion at 1p13.3 covering the gene *GSTM1* (*P* = 0.02, 37.2% cases, 19.5% controls), but downstream quality control by manual inspection of the copy number intensity histogram at this locus, and application of histogram-based association analysis (Barnes et al., 2008) suggested a false positive finding (nominal *P* = 0.26, 51.3% cases, 58.2% controls). A histogram of the copy number signal showed three distinct clusters with 51, 40, and 8% of the cases, respectively, and 58, 37, and 5% of the controls. The three clusters were assumed to correspond to copy numbers of 1, 2, and 3. Further, the deletion allele frequency at this locus has been estimated to be ∼40% in the International HapMap Phase 3 population study (Altshuler et al., 2010).

Given the varying deletion frequencies reported, and to assess the quality of the array-data CNV calling, we performed targeted sequencing of the *GSTM1* (chr1p13) gene in 62 patients. The sequencing data showed three clusters with 52, 40, and 8% of the samples, and 55 out of the 62 patients were present in the same clusters as in the array-data. The CNV frequencies of the three clusters were in perfect agreement with those from the histogram analysis of the array-data, thereby corroborating that deletion of *GSTM1* had no association to TGCC. The sequencing also revealed that the actual copy numbers were 0, 1, and 2 rather than 1, 2, and 3 since the majority of samples had no DNA present (0 sequence reads). The reason for that mis-assignment is that a copy number of 2 is generally assumed by CNV calling software to be the normal copy number state, having the major allele frequency, whereas in this case only 5–10% of the population carry two copies of the gene.

### CNV burden association analysis

Testing whether individuals with TGCC had a greater genomic burden of rare CNVs than controls, we observed a weak indication of increased burden with respect to the number of CNVs per sample, the number of affected genes per sample, and the average length of CNVs per sample (case/control ratio: 1.08, 1.10, and 1.11 respectively), and a significant difference with respect to the total length of all CNVs per sample (case/control ratio: 1.19, *P* = 0.03; Table 1).

**Table 1:** **Global burden of rare CNVs in cases versus controls**.

Type	Burden	*P*	Case:control ratio	Baseline (control)	Baseline (case)
All	Rate	0.09	1.08	4.8	5.17
	Gene rate	0.19	1.10	3.3	3.7
	Mean length (kb)	0.14	1.11	76.9	85.1
	Total length (kb)*	0.03	1.19	372.5	444.3
Duplications only	Rate	0.36	1.03	1.9	1.93
	Gene rate	0.29	1.09	1.9	2.1
	Mean length (kb)*	0.01	1.33	112.3	149.0
	Total length (kb)	0.07	1.22	279.4	340.8
Deletions only	Rate	0.09	1.12	2.9	3.24
	Gene rate	0.23	1.11	1.4	1.6
	Mean length (kb)	0.49	1.00	49.9	49.9
	Total length (kb)	0.21	1.09	151.6	165.0

*Significant difference (*P* < 0.05). Genome-wide *P*-values were estimated by 10,000 permutations of case-control status.

### Gene association analysis

**Figure 2:**
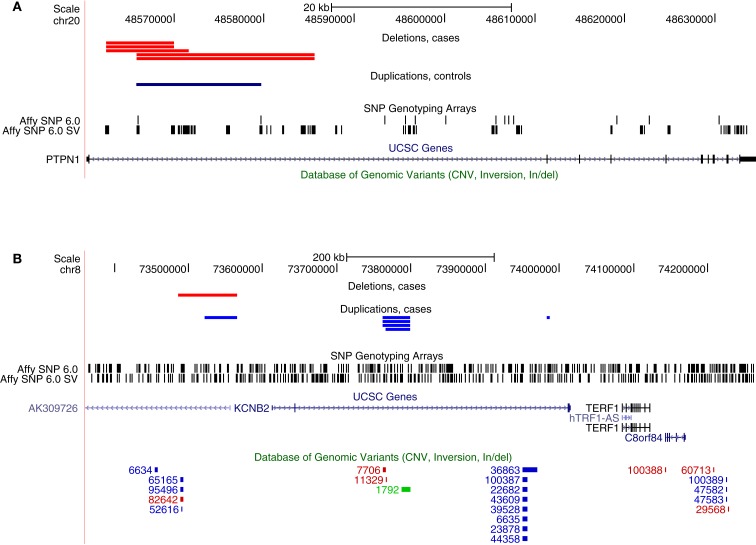
**Genes with a significant excess of rare CNVs among cases as inferred from the genome-wide analysis of array-data**. **(A)** Five cases with deletions and one control with a duplication at an intron of *PTPN1*, **(B)** Six cases with deletions and one case with a duplication at introns of *KCNB2*. The bottom track (Database of Genomic Variants) indicates that many CNVs have previously been observed at *KCNB2* in healthy individuals, whereas none has been observed at *PTPN1*.

Next, we explored if there were any specific genes where rare CNVs were more common in cases than controls. This analysis did not require that CNVs found in different samples overlapped each other, rather, it was sufficient that they were located within the same genic region. Two genes were found to have genome-wide significance, *PTPN1* (*P*_emp._ = 0.038) and *KCNB2* (*P*_emp._ = 0.022), affecting 0.9 and 1.2% of cases, respectively, whereas no occurrence in controls was observed at these loci (Table 2). The CNV at *PTPN1* involved five cases, all found to have a heterozygous deletion at the same intronic region (Figure 2). CNVs at *KCNB2* were found at three different loci: four and one deletions at two different introns and one deletion and one duplication at the promoter (Figure 2). Several CNVs have previously been reported in healthy individuals at *KCNB2*, but not at *PTPN1* (Database of Genomic Variants, v. 10), corroborating a true TGCC association at *PTPN1*, but weakening the possibility of an actual association to *KCNB2*.

**Table 2:** **Genes with an association of rare CNVs**.

Gene	*P*^1^_emp._	*P*_unadjusted_	Odds ratio	Cases (%)	Controls (%)
*PTPN1*	3.8 × 10^−2^	3.9 × 10^−3^	12.31 (1.48–568.17)	0.9	0.0
*KCNB2*	2.2 × 10^−2^	4.1 × 10^−4^	16.58 (2.19–738.20)	1.2	0.0

*^1^Empirical genome-wide *P*-values were estimated by 1,000 permutations of case-control status using Fisher’s test*.

We attempted to verify the CNV at *PTPN1* by performing qPCR on the affected samples, but all five samples with an indication of a heterozygous deletion in the array-data were observed to have two copies in the qPCR. Thus, the CNV calls from the array-data of *PTPN1* are likely to be false positives and no association between *PTPN1* and TGCC can be inferred at this stage.

### Pathway association analysis

Proteins tend to act in concert and perturbations of different components in a set of proteins that are interacting in a network may result in a dysregulation with similar outcome (Lage et al., 2007; Navlakha and Kingsford, 2010; Pers et al., 2011). We therefore conducted association analysis on the level of pathways and protein–protein interaction networks. Association was assessed in the same fashion as for genes and loci described above, that is, by comparing case and control cohorts with respect to the total frequency of rare CNV events targeting a pathway. We compiled comprehensive collections of gene sets, where a set of genes either share function or operate in the same pathway, and performed one thousand random permutations (shuffling case and control status) to estimate a local false-discovery rate (FDR) of each gene set (Efron and Tibshirani, 2002). We observed a significant differential proportion of rare CNVs between cases and controls in 14 gene sets (FDR < 5%, Table 3). However, 11 of these associations were driven by the gene-specific association to *PTPN1* described above. The three remaining gene sets, which did not include *PTPN1*, were: “regulation of cell migration” [go:0030334, 1.8% versus 1.1%, FDR = 0.021, OR = 3.47 (1.12−11.82)], “positive regulation of catalytic activity” [go:0043085, 1.4% versus 0.5%, FDR = 0.04, OR = 5.54 (1.31 − 32.78)], and “macromolecular complex disassembly” [go:0032984, 2.3% versus 1.9%, FDR = 0.047, OR = 2.47 (1.00−6.23)]. The most significant gene set among all sets tested was “regulation of cell migration.” A total of 16 individuals harbored CNVs that altogether overlapped 14 genes in this pathway, at 13 unique CNV loci (Table 4). Genes in this gene set that were affected in cases but not controls included: *BCL2*, *CDH13*, *CORO1A*, *KDR*, *MUC2*, *MUC5AC*, *ONECUT2*, and *PTPRK*.

**Table 3:** **Gene sets with an association of rare CNVs**.

Gene set type^1^	Gene set term	Posterior^2^	Local FDR	Odds ratio	Cases (%)	Controls (%)
GO BP	Regulation of cell migration	0.98	0.021	3.47 (1.1–11.8)	1.8	1.1
GO BP	Macromolecular complex disassembly	0.96	0.040	5.54 (1.3–32.8)	1.4	0.5
GO BP	Positive regulation of catalytic activity	0.95	0.047	2.47 (1.0–6.2)	2.3	1.9

*^1^Many sources of gene sets were jointly analyzed but only sets of the type “gene ontology biological process” were significant, apart from gene sets that included *PTPN1*, which were excluded from the table*.

*^2^The empirical Bayes analysis of microarrays (EBAM) algorithm with 1,000 permutations was used to estimate a posterior and local false discovery rate (FDR) for every gene set*.

**Table 4:** **CNVs targeting genes that are part of the gene set “Regulation of cell migration”**.

CNV	Length^1^	Copy number	Sample	Class	Genes
chr4:55607652…55616597	9	1	165855	Case	*KDR*
chr6:128485528…128525520	40	1	232996	Case	*PTPRK*
chr6:128864684…128871092	6	1	190037	Case	*PTPRK*
chr11:1094626…1140711	46	3	210711	Case	*MUC2, MUC5AC*
chr16:29474810…30099408	625	1	124873	Case	*CORO1A*
chr16:29488112…30085920	598	1	233682	Case	*CORO1A*
chr16:82119367…82175095	56	1	224567	Case	*CDH13*
chr16:82408574…82502970	94	1	232030	Case	*CDH13*
chr18:52763504…53341297	578	3	203688	Case	*ONECUT2*
chr18:59018003…59031365	13	1	231734	Case	*BCL2*
chr2:55119289…56699138	1580	3	M3088A	Control	*RTN4*
chr3:188880305…188936673	56	3	M1270A	Control	*SST*
chr12:50532205…50579767	48	3	M3576A	Control	*ACVRL1*
chr15:50811752…50882082	70	3	M3047A	Control	*ONECUT1*
chr15:97371582…97730964	359	3	M053A	Control	*IGF1R*
chr19:49893892…50298979	405	3	M3381A	Control	*APOE*

*^1^Kilobases*.

## Discussion

We assessed the effect of common and rare CNVs in a TGCC case-control cohort. No single locus was found to be associated to TGCC, but one potential gene network of interest with a weak significant association was identified, having an elevated frequency of rare CNVs among cases. The absence of any single locus CNV associated to TGCC is in line with the relatively few findings for other diseases, including a screening of ∼3,400 common CNVs in eight common diseases (Craddock et al., 2010). Furthermore, common CNVs are typically ancient variations, which are tightly correlated with single nucleotide polymorphisms (SNPs), and can therefore be detected by genome-wide association studies of common SNPs (Lander, 2011). However, one should not neglect the importance of common CNVs in gene-phenotype association studies, since there exists evidence that disease associated SNPs have a tendency to tag CNVs more often than random, and that such CNV-tagging SNPs are enriched for expression quantitative trait loci (eQTL; Gamazon et al., 2011). Further, rare CNVs are not tagged by common SNPs implicated by GWASs and they have been found to play a major role in neurodevelopmental disorders (Merikangas et al., 2009) and rare *de novo* CNVs were recently found in 3 out of 43 TGCC family-trios (Stadler et al., 2012). We have previously searched for CNVs associated to familial TGCTs, where the inheritance component is much higher than among sporadic cases, but failed to find a CNV that was significant across the small set of studied families. We only identified a handful of CNVs that segregated with TGCT, but these were either family-specific or relatively common variants, such as *RLN1* (Edsgärd et al., 2011).

The rare association analysis presented in this study initially indicated two genes to be associated with TGCC, *PTPN1*, and *KCNB2*. *KCNB2* was considered a false positive due to the amount of previously reported CNVs in independent control cohorts at this locus, being on par with that of the frequency in the case group of this study. *PTPN1* appeared as a good candidate since it has previously been implicated with the progression of prostate cancer along with evidence that the androgen receptor is a transcriptional regulator of *PTPN1* (Lessard et al., 2010, 2012). A loss of *PTPN1* may thereby be associated with impaired responsiveness to androgens, which would be consistent with the fact that low androgen status during development is a risk factor for TGCT (Rajpert-De Meyts and Skakkebaek, 1993; Sonne et al., 2008). Further, CNVs at the 20q13 chromosomal region have been observed in several other cancers (Nishizaki et al., 1998; Schaid, 2004; Furukawa et al., 2006). Furthermore, all affected probands in this study presented a deletion, consistent with a tumor suppressing function of oncogenic kinases, and *PTPN1* has been shown to be able to play both a pro- and anti-oncogenic role (Stuible et al., 2008). A considerable effort was made to ensure high quality of the CNV calls: by using two complementary CNV calling methods, by applying several strict QC criteria, and by manual inspection of the raw probe signals. However, despite the stringent QC the qPCR did not verify the heterozygous deletion of *PTPN1* in any of the samples. This highlights the difficulty of assessing the reliability of CNV calls from genome-wide short-nucleotide microarrays.

Our pathway analysis identified the gene set “regulation of cell migration” as having the highest difference in proportion of rare CNVs in cases compared to controls. There is a growing body of information that strongly suggests a crucial role of primordial germ cell (PGC) biology in TGCC oncogenesis. PGCs are embryonic cells which during mid-gestation migrate from the base of the yolk sac, along the hind-gut, to the genital ridge, one of the longest migrations of all mammalian cells. Four recent SNP GWAS TGCC studies associated *KITLG* and a number of other genes related to the *KIT-KITLG* pathway (Kanetsky et al., 2009; Rapley et al., 2009; Turnbull et al., 2010; Dalgaard et al., 2012), a regulatory network which is believed to be of crucial importance in the determination of the fate of PGCs (Rapley and Nathanson, 2010). For instance, mutations of the KIT receptor, or the KIT ligand, in the mouse, blocks PGC migration, resulting in infertility (Matzuk and Lamb, 2002). In addition, a disturbance of the migration of PGCs during early fetal development may cause extragonadal germ cell cancers along the midline of the body (Oosterhuis and Looijenga, 2005). One of the afflicted genes in the “regulation of cell migration” gene set was *PTPRK*, at which two samples had a deletion. Like *PTPN1*, *PTPRK* belongs to the family of protein tyrosine phosphatases, but it is a membrane-bound receptor. *PTRPK* has been implicated in TGFβ-signaling (Xu et al., 2010), and a recent GWAS study indicated the involvement of TGFβ superfamily signaling in testicular dysgenesis (Dalgaard et al., 2012). In mouse, PGCs divide rapidly under the influence of TGFβ signaling factors, and defects in PGC development is observed in knockout models of bone morphogenetic proteins (BMPs; Lawson et al., 1999; Ying et al., 2000). In total there were 14 genes which were part of the “regulation of cell migration” pathway and that harbored CNVs. It would be of high interest to further elucidate the role of these genes by studying how the CNVs affect the expression levels of the corresponding genes. What may complicate matters is if the critical period of time is the development of the fetus, which may force one to revert to the study of animal models.

In the analysis of common CNVs we made a further assessment of the *GSTM1* CNV frequency by targeted sequencing. The main reason for this was to assess the quality of and verify the array-data CNV calls of a seemingly problematic locus. However, *GSTM1* was also partly chosen due to that the risk of developing TGCC has been shown to increase by the exposure to certain environmental factors (Skakkebaek et al., 2001) and *GSTM1* is known to be involved in the detoxification of xenobiotic compounds (Hengstler et al., 1998). Hypothetically, it is not unlikely that there exists a genetic variation that may affect the ability to metabolize environmental chemicals, which in turn affects the risk of TGCC. Apart from such gene-environment interactions it is probable that part of the unexplained heritability can be explained by gene–gene interactions, where a combination of several genetic variations cause a greater effect on the phenotype than the sum of their individual effects (epistasis). Further research is required to assess the effect of rare variants in varying genetic backgrounds.

In conclusion, this study corroborates the rejection of the hypothesis that a single CNV locus mediates a major risk for TGCC. It suggests that several rare CNVs may contribute to the oncogenesis of a subset of TGCC subjects, but the frequency after aggregation of CNVs on the implicated gene and pathways is still low, and these CNVs therefore only provide a minor contribution to the overall heritability. Larger cohorts are needed to further explore the impact of rare variants in TGCC.

## Materials and Methods

### Sample collection

Two hundred and twelve men with TGCCs and 439 healthy young men with semen concentrations above 60 million sperm/ml were collected at the Department of Growth and Reproduction, Rigshospitalet, Denmark. All DNA samples were obtained from men of Danish ancestry, who provided informed consent for genetic analysis. The samples were coded during the entire analysis. The project has been approved by the Regional Medical Ethics Committee (Nr H-KF-265848) and the Danish Personal Data Protection Agency (Nr 2008-41-2158). This cohort has previously been analyzed with respect to single nucleotide variation (Dalgaard et al., 2012). Sixty-two of the TGCC patients were selected for sequencing of the *GSTM1* gene.

### Genotyping and SNP-based sample quality control

Germline DNA was extracted from peripheral blood using QuickGene DNA whole blood kits from FujiFilm Life Science according to the manufacturer’s manual. Samples were genotyped using the Affymetrix Genome-wide Human SNP Array 6.0. Here we present the analysis of CNVs, SNP association analysis and details of the cohort are described elsewhere (Dalgaard et al., 2012), but an initial sample quality control was performed using SNP genotypes called by the Birdseed algorithm [Affymetrix Power Tools (APT) v. 1.10.2]. We excluded samples based on a genotyping call rate below 96%; QC contrast below 0.4 as according to the Affymetrix GCOS software; non-European ancestry by inspection of a plot of the first two principal components of the cohort and the HapMap phase III samples (Altshuler et al., 2010); high degree of relatedness based on identity-by-descent where one individual was kept among related samples; and samples with an outlying inbreeding coefficient.

### CNV detection and quality control

For samples that passed the SNP quality control described above we ran two CNV calling algorithms, BirdSuite (v. 1.5.5) and PennCNV (v. 2010May01). PennCNV requires a signal intensity file and a SNP genotyping file and these were generated by quantile normalization of PM-only probes with median polish probe summarization (APT v. 1.12.0) and Birdseed (v. 2 in APT 1.12.0), respectively. We excluded CNVs which failed any of the four following criteria: (1) A CNV was not called by both algorithms. A histogram of the percentage of overlap indicates that the vast majority has >90% overlap, but we set the threshold to at least 10%. (2) A CNV log odds confidence score larger than two, as recommended by BirdSuite (Korn et al., 2008). (3) CNV size was less than five markers or four kilobases, in effect excluding the 25% short-length quantile of CNVs. (4) A CNV was longer than one megabase (three outliers based on histogram). Further, samples were excluded with respect to the three following criteria: (1) extreme sample burden in terms of more than 110 CNVs (four outliers); or (2) a total length of CNVs larger than 7.5 megabases (two outliers); (3) bad sample quality in terms of high variance of copy number signal (median copy number variance larger than two, as recommended by BirdSuite, or a Log R Ratio standard deviation (LRR SD) obtained from PennCNV larger than 0.4). LRR SD was set according to PennCNV guidelines when CNV calling are performed on Affymetrix samples. Finally, rare CNVs that had more than 50% overlap with segmental duplication regions (retrieved from UCSC hg18) were removed, since such regions have been shown to generate more false CNV calls (Pinto et al., 2010). A total of 189 TGCC cases and 380 controls remained after the completion of all quality control steps, harboring a total of 1008 and 1872 rare CNVs, respectively.

### CNV association analysis

Copy number variant association analysis was performed using plink (v. 1.07) and custom R (v. 2.12) scripts. Common and rare CNVs were defined as those with an allele frequency above or below 1%, respectively. The allele frequency of a CNV was determined using the locus within a CNV region with the maximum number of overlapping individual CNVs.

### Locus association

Common CNVs were evaluated by searching the whole genome for loci with a significantly higher degree of affected cases as compared to controls. Binary state frequencies were used, and a Fisher test was performed for deletion, amplification, and any type of aberration, respectively. Genome-wide significance was estimated by generating a null distribution based on one thousand case-control status permutations. For each permutation the minimal *P*-value was selected, thereby providing control of the family-wise error rate (FWER). Loci with significant associations were further verified by CNVtools (Barnes et al., 2008) which uses a complementary CNV calling strategy, since it employs a statistical model based on density-based clustering rather than a hidden Markov model. Furthermore, loci residing at the edges of a common CNV and associations from variation of boundary truncation were excluded.

### Global burden analysis

The impact of rare CNVs was assessed by three approaches: genome-burden analysis, gene association, and pathway association analysis.

The global burden of rare CNVs in cases compared to controls were assessed with respect to (i) the number of CNVs per sample, (ii) the number of affected genes per sample, (iii) the average length of CNVs per sample, and (iv) the total length of CNVs per sample.

### Gene association

Gene association analysis was performed using the number of case and control samples harboring a rare CNV that overlapped the gene of interest. Genes were retrieved from UCSC (hg18). CNV frequencies were compared with Fisher’s test and multiple testing corrected *P*-values were obtained based on case-control permutation as described above for the locus association of common CNVs. Significant CNVs which were found to have a lower allele frequency in our case cohort than in the Database of Genomic Variants (DGV, v. 10), were considered false positives.

### Pathway association

Pathway association analysis was performed based on the number of case and control samples that had a rare CNV in any of the genes of a pathway. The R package “siggenes” was used to obtain *P*-values corrected for multiple testing across all tested gene sets. The package provides a FDR, based on randomized case-control sampling, as well as an adjustment of the variance of an individual pathway using information from the observed variances of all pathways (Efron and Tibshirani, 2002). Gene sets were retrieved from KEGG (Kyoto Encyclopedia of Genes and Genomes; Ogata et al., 1999), Reactome (D’Eustachio, 2011), BioCarta^1^, NCI-Nature curated pathways (Pathway Interaction Database; Schaefer et al., 2009), GO (Gene Ontology; Ashburner et al., 2000), COSMIC (Catalog of Somatic Mutations In Cancer; Forbes et al., 2011), Cyclebase (Gauthier et al., 2010), protein–protein interaction complexes (Lage et al., 2007), OMIM (Online Mendelian Inheritance in Man^2^), MGI (Mouse Genome Informatics^3^) and a set of candidate infertility genes from a recent review (Matzuk and Lamb, 2008). Terms annotating more than 700 or less than 5 genes were discarded, since they do not produce meaningful statistical results.

### Verification of PTPN1 copy number

Copy number verification of PTPN1 was done using Quantitative PCR on the Mx3000P platform from Stratagene (Agilent Technologies, Santa Clara CA, USA). The protocol has been described previously (Ottesen et al., 2007). Primers for the CNV at PTPN1 were designed using Primer3 (Rozen and Skaletsky, 2000). Primer sequences for PTPN1 were: forward 5′-TTC AAC CCT AAC TAG GTA TGC A-3′ and reverse 5′-CTA AAA TGC TGG AGA CTT AGG T-3′ and primers for GAPDH: forward 5′-CTC CCC ACA CAC ATG CAC TTA-3′ and reverse 5′-TTG CCA AGT TGC CTG TCC TT-3′ (DNA Technology A/S, Aarhus, Denmark). GAPDH was used as endogenous control gene. Mixtures of forward and reverse primers were denatured for 3 min at 95°C and incubated on ice until use. DNA concentrations were 8–17 ng, 15 μL Brilliant SYBR Green QPCR Master Mix (Stratagene), 7.0 μL primer mixture of PTPN1 (final conc.: Fw and Rev 100 nm) or GAPDH (final conc.: Fw and Rev 100 nm), and a total volume of 30 μL. Conditions for amplification were as follows: one cycle at 95°C for 10 min and 40 cycles at 95°C for 1 min, 62°C for 1 min and 72°C for 1 min. The PTPN1:GAPDH ratio was calibrated to a normal male reference control of DNA, as previously described for other genes (Ottesen et al., 2007; Mau Kai et al., 2008). All specimens were analyzed in triplicate and the mean-ratio was used to infer integer copy number.

### Targeted sequencing of GSTM1

#### Design of targeted capture baits

Target capture was designed on the Agilent SureSelect capture system (Santa Clara CA, USA). Baits for capture were designed by tiling the genomic region harboring *GSTM1* with an approximately 50% overlap between consecutive baits. The bait sequences were optimized to avoid regions with extreme GC content or highly variable regions, and to avoid possible cross-hybridization or self-folding of the baits, which could decrease the hybridization specificity. The principle of this method has been described previously (Wesolowska et al., 2011).

#### Library preparation, pooling, target enrichment, and sequencing

DNA shearing, library preparation, and pooling were performed using a modified protocol of the SureSelect Target Enrichment System (Agilent Technologies) to allow sample multiplexing as described elsewhere (Wesolowska et al., 2011). Three micrograms of genomic DNA was sheared by ultrasound (Covaris; Woburn, MA, USA) to yield fragments of an average size of ∼200 bp. The sheared fragments were ligated with custom made adaptors containing a unique four-base barcode, and subsequently purified and amplified by ligation-mediated PCR (LM-PCR). Amplified DNA was pooled in groups of 10 with equal amount of each, after which enrichment was performed by hybridization to custom SureSelect target capture baits (Agilent Technologies). The captured libraries were processed with Illumina Cluster Generation Station (Illumina, San Diego CA, USA) following the manufactures recommendations. We performed 100 nt single-end read sequencing on the Illumina HiSeq 2000 sequencing platform.

#### Data analysis

Sequence reads were trimmed and high quality reads were mapped to the human reference genome build 37 (GRCH37) using Burrow-Wheeler Alignment algorithm (Li and Durbin, 2010). Alignments with mapping score below 30 were discarded and PCR duplicates were removed with Picard MarkDuplicates^4^. Copy numbers were estimated by calculating a sequence depth ratio from the number of reads in the targeted genomic region, normalized by size of the region and total number of reads for the sample. A histogram of the sequence depth ratio from all individuals identified three distinct clusters corresponding to two copies and heterozygous and homozygous deletions.

## Conflict of Interest Statement

The authors declare that the research was conducted in the absence of any commercial or financial relationships that could be construed as a potential conflict of interest.

## Footnotes

^1^http://www.biocarta.com

^2^http://www.ncbi.nlm.nih.gov/omim

^3^http://www.informatics.jax.org

^4^http://picard.sourceforge.net
